# A rapid and sensitive UPLC-MS/MS method for the determination of flibanserin in rat plasma: application to a pharmacokinetic study

**DOI:** 10.1186/s13065-019-0620-9

**Published:** 2019-08-27

**Authors:** Long He, Wenting You, Sa Wang, Tian Jiang, Caiming Chen

**Affiliations:** 1 Clinical Laboratory, The Affiliated Wenling Hospital of Wenzhou Medial University, Wenling, 317500 China; 2 Department of Pharmacy, The Affiliated Wenling Hospital of Wenzhou Medial University, No 190 Taiping South Road, Wenling, 317500 Zhejiang China; 3 Neurology Department, The Affiliated Wenling Hospital of Wenzhou Medial University, Wenling, 317500 China

**Keywords:** Flibanserin, Rat plasma, UHPLC-MS/MS, Pharmacokinetics

## Abstract

**Background:**

In this work, we aim to develop and validate a fast, simple, and sensitive method for the quantitative determination of flibanserin and the exploration of its pharmacokinetics.

**Methods:**

Ultra-performance liquid chromatography-tandem mass spectrometry (UHPLC-MS/MS) was the method of choice for this investigation and carbamazepine was selected as an internal standard (IS). The plasma samples were processed by one-step protein precipitation using acetonitrile. The highly selective chromatographic separation of flibanserin and carbamazepine (IS) was realised using an Agilent RRHD Eclipse Plus C18 (2.1 × 50 mm, 1.8 µ) column with a gradient mobile phase consisting of 0.1% formic acid in water and acetonitrile. The analytes were detected using positive-ion electrospray ionization mass spectrometry via multiple reaction monitoring (MRM). The target fragment ions were m/z 391.3 → 161.3 for flibanserin and m/z 237.1 → 194 for carbamazepine (IS). The method was validated by linear calibration plots over the range of 100–120,000 ng/mL for flibanserin (R^2^ = 0.999) in rat plasma.

**Results:**

The extraction recovery of flibanserin was in the range of 91.5–95.8%. The determined inter- and intra-day precision was below 12.0%, and the accuracy was from − 6.6 to 12.0%. No obvious matrix effect and astaticism was observed for flibanserin. The target analytes were long-lasting and stable in rat plasma for 12 h at room temperature, 48 h at 4 °C, 30 days at − 20 °C, as well as after three freeze–thaw cycles (from − 20 °C to room temperature). The proposed method has been fully validated and successfully applied to the pharmacokinetic study of flibanserin.

## Introduction

Hypoactive sexual desire disorder (HSDD) is defined as a disease that results in the recurrent or persistent absence or deficiency of desire for sexual activity and sexual fantasies, which results in pronounced distress or interpersonal difficulty [1]. In the past, due to traditional societal and cultural beliefs as well as other reasons, research into female sexual dysfunction has persistently been neglected. Many large-scale studies have determined that approximately one-third of premenopausal women in the US experience distress over their sexual relationships, and the incidence of HSDD continues to rapidly increase [2, 3]. An imbalance of biologic factors, which are responsible for inhibitory and excitatory activity, is thought to be the primary reason for the misregulation of sexual responses in the central nervous system (CNS), resulting in sexual dysfunction [4]. Using positron emission tomography and FMRI scans, Stahl et al. reported that compared to normal women, those with HSDD demonstrated lower activity in certain cortical and limbic areas of the brain when viewing pornographic material [5]. This indicated that the source of HSDD is a biological dysfunction within the brain. Studies on animals have also investigated the sexual side effects of certain medications that affect the serotonergic and dopaminergic systems. They showed that excessive serotonin (5-HT) activity and subnormal noradrenergic and dopaminergic receptor activity or function may inhibit sexual desire and result in HSDD. Dopamine (DA) and Norepinephrine (NE) are thought to be major ‘initiators’ of sexual arousal and modulators of sexual excitement [6]. Hence, in order to restore a balanced and healthy sexual response, a modulation of these factors is required.

**Fig. 1:**
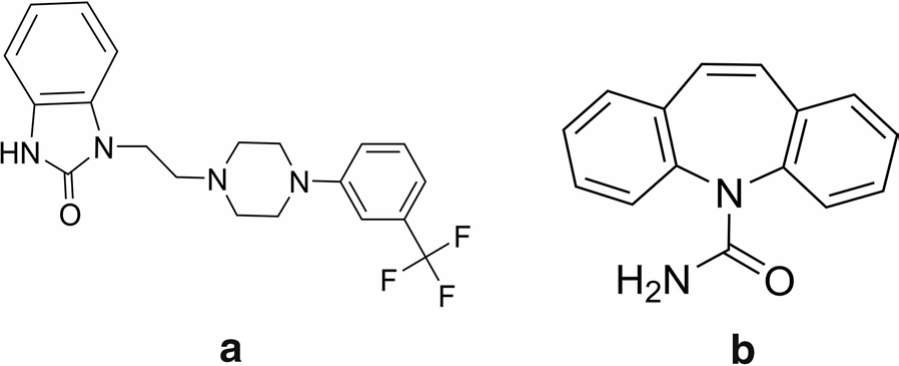
The chemical structures of flibanserin and IS in the present study: **a** flibanserin; **b** carbamazepine (IS)

Flibanserin is the first approved drug for the treatment of HSDD, and was developed by Sprout Pharmaceuticals (US) and approved in 2015 (Fig. 1) [7]. Flibanserin is a non-hormonal oral medication that affects neurotransmitter levels in the CNS, leading to their normalization and restoration of sexual function. In previous research, it was demonstrated that flibanserin behaves as a 5-HT2A antagonist, and a 5-HT1A agonist, as well as having an affinity to 5-HT2B, 5-HT2C, and the dopamine receptors of D4 [8] Flibanserin inhibits serotonin and elevates the number of dopamine receptors. This is believed to promote dopaminergic effects while inhibiting ‘anti-sexual’ serotonergic effects, which is associated with enhanced sexual desire. In addition, serotonin can exert an inhibitory influence on adrenaline, so reducing the levels of serotonin would promote the levels of norepinephrine, which can also activate sexual desire. Katz et al., Thorp et al., and Rosen et al. reported three clinical phase III double-blind trials with 2310 premenopausal women suffering from HSDD. For the trial, 1238 participants received daily treatment with a placebo before sleep and the rest were treated with 100 mg of flibanserin. After 24-weeks treatment, the flibanserin group showed a remarkable enhancement in both clinic and statistical data associated satisfactory sexual events and level of arousal compared with the placebo group [9–11]. During the clinical trial, the rate of serious adverse events (SAEs) was ≤ 0.9%, and it was thought that these SAE did not have a relation to the flibanserin treatment. The common adverse events (AEs) reported by patients with flibanserin were hypotension, syncope, and somnolence [9–11].

Flibanserin can cause severe hypotension and syncope when patients drink alcohol or take flibanserin with moderate or strong CYP3A4 inhibitors [12]. This is due to an interference with the metabolism of flibanserin in the body caused by these factors. Many studies have been conducted on the risks associated with the interaction between flibanserin and alcohol, and its interaction with moderate or strong CYP3A4 inhibitors; however, there is minimal research on the pharmacokinetics and quantitative determination of flibanserin. Magdalena et al. reported an approach using LC–MS/MS to determine flibanserin in organic solvents [13]. To the best of our knowledge, there are no published reports on the determination of flibanserin in plasma. In the present work, the objective was to formulate a recognized and sensitive method to characterize flibanserin’s plasma pharmacokinetics. The broader goal being to use this knowledge to prevent adverse effects and maximize its therapeutic effects. In addition, using UHPLC-MS/MS, we developed a method to detect the pharmacokinetic properties of flibanserin. The method was demonstrated to be selective, linear, precise, stabilized, and was successfully applied for the quantification of flibanserin in rat plasma.

## Materials and methods

### Chemicals and reagents

We purchased flibanserin (over 98% purity) from perfemiker (Shanghai, China). The carbamazepine (purity > 98%) was acquired from Sigma-Aldrich (St. Louis, MO, USA). As HPLC grade, the methanol, formic acid and acetonitrile were bought in Merck Company (Darmstadt, Germany). In addition, the ultrapure water was obtained from the Milli-Q Reagent water system (Millipore, MA, USA).

### Instrumentation and conditions

We conducted the samples analysis by chromatographic system of Agilent 1290 of ultra-performance liquid chromatography (UHPLC, Agilent Technologies, Santa Clara, CA, USA) coupled to an Agilent 6490 Triple Quadrupole mass spectrometer (Agilent Technologies), which possessed the triple quadrupole mass spectrometer, a degasser, a HiP sampler, a column compartment and a binary pump. The column of RRHD Eclipse Plus C18 (2.1 × 50 mm, 1.8 µ) at the constant temperature of 35 °C was applied to the separation of compounds. The optimal choice of phase of mobile comprised acetonitrile (B) and formic acid (A) of 0.1%. Gradient elution’s course was utilized in the following: linear increase for 0–1.0 min to 90% of B, 1.0–2.6 min maintained at 90%, 2.6–3 min linear decrease to 20% of B. Injection of analyte volume was 2 µL and flow rate of mobile was 0.4 mL/min. Entire run time of the whole elution procedure was 4.5 min, which included one program of gradient elution program for 3 min with one post time for 1.5 min. Under above liquid phase conditions, the target analyte of flibanserin and IS were completely partition. Besides, the retention time of them were 2.51 and 2.25 min, respectively.

Sample testing were adopted by the Agilent 6490 Triple Quadruple LC/MS. The source of electrospray ionization (ESI) was offered to the system, and quantification was conducted in a positive mode. In the positive mode, we set the capillary voltage to 4.0 kV, set nebulizer to 45 psi, and the flow of gas to 10 L/min at the temperature of 350 °C. In addition, we utilized the MassHunter Workstation to obtain data and the Qualitative Analysis software (version B.07.00) was used to data analysis. The detection of specific parent ion and product ions (qualifier and quantifier) of the flibanserin and IS was operated in one dynamical approach of multiple reaction monitoring (MRM) in their time window of retention. In order to assure the specificity of detection of flibanserin and IS, except the particular transition of MRM of analyte, respective retention time, quantifier’s ratio and the ratio of product ion of qualifiers were all considered. In addition, we used the most intensive fragment as the quantifier, and used the second on for qualification in order to assure the specific detection. Table 1 showed the details of parameters of MS of flibanserin as well as IS.

**Table 1:** MS parameters of flibanserin and carbamazepine

Compound name	Precursor ion (m/z)	Product ion 1 (m/z)	Collision energy 1 (V)	Product ion 2 (m/z)	Collision energy 2 (V)	Fragmentor
Flibanserin	391.3	161.3	25	119.1	30	170
Carbamazepine	237.1	194	18	193.1	38	140

### Preparation of calibration standards and quality control samples

Stock solution of 10 mg flibanserin was dissolved in 10 mL of methanol, and 1.0 mg/mL of concentration were obtained, which was utilized to generate the calibration standard as well as the quality control (QC) samples. Working solutions of flibanserin were prepared by the means of diluting the level of corresponding stock solutions to several respective levels of concentration. Both calibration standards of flibanserin and QC samples were made using suitable working solutions with the blank plasma of rat. We prepared the plots of calibration by adding 10 µL of corresponding working solution into 90 µL of blank plasma of rat. The ultimate concentrations of calibration standards of flibanserin were 100, 500, 2500, 5000, 10,000, 20,000, 40,000, 80,000, 120,000 ng/mL, respectively. Moreover, we prepared the three respective levels of QC samples independently in a similar manner, which were considered as calibration: LQC (800 ng/mL), MQC (8000 ng/mL), and HQC (80,000 ng/mL). Stock solution of IS was processed by dissolving 10 mg of carbamazepine into 10 mL of methanol to 1.0 mg/mL of ultimate concentration. Working solution of IS (10,000 ng/mL) was processed by diluting corresponding stock solution by utilizing methanol. All of the stock solutions, QC samples, working solutions and calibration standards were stored at the temperature of − 20 °C until analysis.

### Sample preparation

For each 1.5 mL centrifuge tube, 200 µL acetonitrile were added to 100 µL thawed plasma samples for protein precipitation, then 30 µL IS (10,000 ng/mL) was added. We mixed the tubes in vortex for 2 min to blend fully, and centrifuged it at 12,000 rpm for 10 min. After gently mixed for 20 s, we pipetted 50 µL of the mixture into a UHPLC vial, and injected 2 µL aliquot of mixture in UHPLC to perform analysis.

### Method validation

According to the validation guidance of bioanalytical method stimulated by the United States Food and Drug Administration (US-FDA, 2001) [14], this method was fully verified to be specific, accurate, precise, linear, recovered, stabilized and not had matrix effect, the flibanserin in the plasma of rat would be determined before adopting the method.

Selectivity is a specialty of a method which can validate that there was no probable interference of endogenous substances with the targeted analyte and IS [15]. The method was evaluated by conducting analysis on six samples of blank plasma (rats are different), there was flibanserin and IS in blank sample, and the samples of rat plasma were obtained after oral administration.

The precision and accuracy of the present method were evaluated by determination of three different concentrations (800, 8000, 80,000 ng/mL) of QC samples in the plasma of rat on 1 day or three consecutive days. Moreover, we utilized RE (relative error, the percentage of concentration measured via the nominal concentration, %) and RSD (relative standard deviation, %) to value the degree of precision and accuracy of method. As required, the variation of accuracy should between − 15 and 15%, and precision should be within 15%.

In order to make assessment on linearity, we processed and analysed the calibration standards of nine diverse flibanserin concentrations (100–12,000 ng/mL) on three separate days, respectively. We assessed the linearity of flibanserin by weighing (1/× 2) linear regression of least square method of the ratios of peak area against these concentrations. We defined LLOQ as the minimum permissible concentration on calibration curves, which were fixed at an acceptable degree of precision and accuracy.

We evaluated Matrix effect (ME) by collecting six samples of blank plasma of several animals. ME was assessed through three QC levels with spiking of the ratio of peak areas of analytes after extraction the blank plasma and peak area of neat standard solutions when they are at corresponding concentrations. The extraction recovery possessed the ability to extract the targeted analyte from these biological samples in test. It was assessed by making comparison between the ratios of peak area of QC samples that were extracted and those of subsequent samples extracted, which contained equal amount of reference QC solutions (n = 6). We evaluated the extraction recovery and ME of IS was evaluated by the same method.

The stability of this method was validated by measurement of six replicates of plasmatic samples at three concentration levels (800, 8000, 80,000 ng/mL) in various handing conditions. QC Samples were analysed in four storage conditions: short-run stability (indoor temperature for 12 h), three freezing–thawing stabilities (ranging from − 20 °C to indoor temperature in three freezing–thawing cycles), medium-term stability (48 h in automatic sampler at 4 °C) and long-term stability (− 20 °C for 30 days). The assay values of precision (RSD% ≤ 15%) and accuracy (RE% ≤ ±15%) within acceptable limits were considered stable [16, 17].

### Pharmacokinetic study

We obtained six male Sprague–Dawley rats (180–220 g) in Experimental Animal Center in Wenzhou Medical University (Wen-Zhou, China). The six rats were injected into oral flibanserin to study pharmacokinetics. All procedures and protocols in this experiment conformed to the Guide for the Care and Use of Laboratory Animals and gained permission by the Animal Care and Use Committee. Before 12 h of experience, the rats were not allowed to diet overnight except water. Furthermore, we suspended flibanserin in 0.5% of Carboxy Methyl Cellulose (CMC), and the dosage of oral administration was 10 mg/kg. After intragastric administration, we put blood samples (300 µL) from caudal vein of rats into 1.5 mL polythene tubes of heparinized at 0.083, 0.25, 0.5, 0.75, 1, 2, 4, 6, 8, 10 and 12 h, respectively. The blood samples were separated immediately at 4000 g for 8 min, then transferred the separated plasma (> 100 µL) into one clean tube, which was not stored at − 80 °C cryogenic refrigerator until analysis was performed. Comparison of concentration of plasmatic flibanserin and time data for every rat the DAS (Drug and Statistics) software (Version 3.0, Shanghai University of Traditional Chinese Medicine in China).

### Euthanasia

After the pharmacokinetic study, all animals were euthanized by carbon dioxide inhalation. Animals were placed one by one in the euthanasia box filled with air, but immediately after placement of the animals, carbon dioxide started to stream into the box with a flow rate of 25% V/min. The gas flow was be maintained for 2 min after animal apparent clinical death [18].

## Results and discussion

### Method development and optimization

The liquid chromatography conditions were investigated with the goal of separating interfering analytes, improving the detection sensitivity and shortening the runtime. This included optimization of the composition and ratios of the mobile phase, and the column and its temperature. The RRHD Eclipse Plus C18 column (2.1 × 50 mm, 1.8 µm) demonstrated good symmetry for the analyte peak and a proper retention time.

In order to achieve effective separation, the peak shape needs to be symmetrical and the retention time should be shortened. A mixture of 0.1% formic acid in water and acetonitrile was used as the mobile phase composition and gradient elution was applied. The flow rate was investigated over a range between 0.2 and 1.0 mL/min and the effect of the column temperature was studied in the range of 20 to 40 °C. For a mobile phase formed with 0.1% of formic acid in water (A) and acetonitrile (B), optimal results could be obtained using a flow rate of 0.4 mL/min and a column temperature of 35 °C. Under these conditions, symmetrical peaks, a high detection of the target analytes, and a shortened retention time were achieved. Measurement of the analytes and IS were performed by gradient elution for 3 min with a post time of 1.5 min. The elution of flibanserin began in the mobile phase with 0.1% formic acid in acetonitrile (20:80, V/V). Then, between 0 and 1.0 min the rate of acetonitrile demonstrated a linear increase to 90%, this percentage was maintained up till the 2.6 min mark. Between 2.6 and 3.0 min, while the acetonitrile concentration was linear, it returned back to a concentration of 20%. Under these conditions, the chromatograms had a symmetric peak shape, good separation and demonstrated an optimal resolution over a short operating time.

We optimized the mass parameters in order to achieve a higher response and better resolution. First, the fragmentor was set in a rough range from 50 to 240, and the Collision Energy (CE) range was between 10 and 50 in the positive mode. After completing the MS/MS optimization procedure, the most intense fragment was used for the quantification of flibanserin and IS, and the second most intense one was used for qualification of the target analytes.

Compared to some other methods, the UPLC method has some advantages; primarily that it could remove potential interferences more efficiently than HPLC [19]. A UPLC-MS equipped with a RRHD Eclipse Plus C18 column demonstrated higher performance than HPLC and could significantly reduce the retention time [20].

### Optimization of sample pre-treatment and IS

Solid phase extraction (SPE), liquid–liquid extraction (LLE) and protein precipitation (PPT) are the three most frequently used methods to prepare biological samples. However, the LLE and SPE are complex, time-consuming and environmentally unfriendly. So PPT was adopted in the study for sample preparation. This method has the benefits of decreasing sample preparation time, has no further concentration procedure and can achieve a high extraction efficiency compared to the other methods. Different organic solvents are generally used for the extraction of target analytes from various tissues, so three PPT solvents (methanol, acetonitrile and acetonitrile-methanol) were tested. The results revealed that acetonitrile could achieve a higher analyte recovery rate (91.5–95.7%) than the other solvents [21]. Precipitation with acetonitrile also led to lower background noise and a higher sensitivity compared to the other solvents. Therefore, we chose a precipitation procedure with acetonitrile to treat the plasma samples.

### Selectivity and ME

**Fig. 2:**
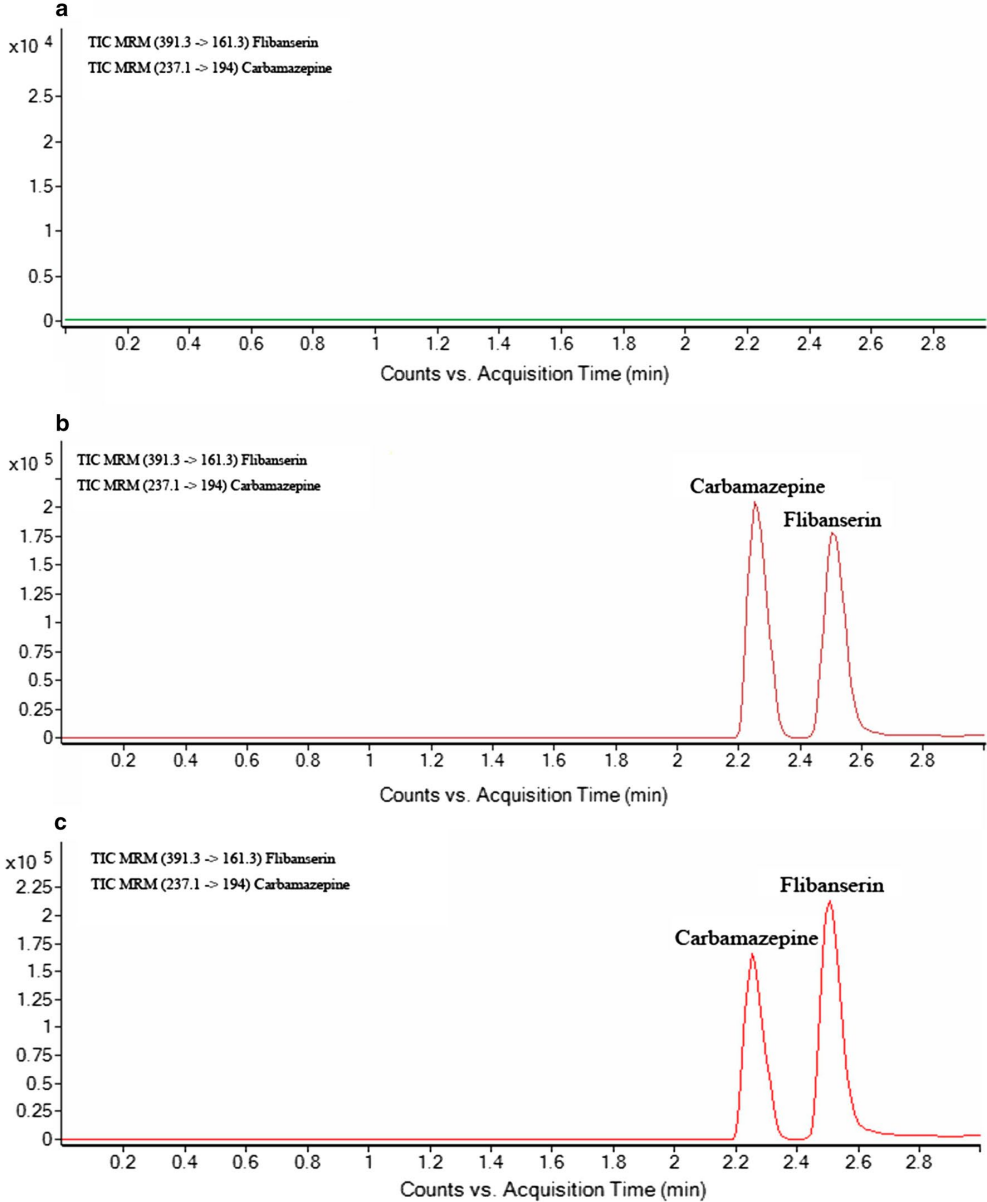
Representative UHPLC-MS/MS chromatograms of flibanserin and carbamazepine (IS). **a** Blank plasma; **b** a blank plasma sample spiked with flibanserin and IS; **c** a rat plasma sample obtained 1 h after oral administration of flibanserin

Using the optimized mass spectrometry and chromatographic conditions, the retention times of IS and flibanserin were 2.25 min and 2.51 min, respectively. Figure 2 demonstrates the chromatograms of blank plasma, blank plasma spiked with flibanserin and a sample of rat plasma. There were no endogenous interfering peaks compared with the blank plasma chromatogram.

The matrix effects of flibanserin at concentrations of 800, 8000, 80,000 ng/mL were 92.0%, 87.8%, 106.3% (n = 6), respectively. The matrix effects of IS at 10,000 ng/mL was 103.5% (n = 6). The results showed there are negligible the matrix effects.

### Calibration curve and sensitivity

Linear regression analysis was carried out for the ratios of the peak area versus their corresponding concentrations. The calibration curve for the nine flibanserin concentrations ranged over 100–120,000 ng/mL, which resulted in a favourable linear relationship with a regression coefficient of R^2^ = 0.999. At the lower limit of quantification (LLOQ) for flibanserin, the values for the accuracy and precision had a 10.9% relative standard deviation (RSD) and a 4.7% relative error (RE), respectively.

### Accuracy and precision

The method’s accuracy and precision were determined by calculating both the RSD and RE for the six QC sample replicates at three concentrations over three separate days. The results for the accuracy and precision of all the QC samples are summarized in Table 2. The RSD and RE were respectively used to illustrate the precision and accuracy. Inter-day and intra-day RSDs were below 12.0%, and the corresponding REs ranged from − 6.6 to 12.0% at each flibanserin concentration in rat plasma. This revealed that the approach used to determine flibanserin was reliable, accurate and reproducible.

**Table 2:** Precision, accuracy, recovery and ME for flibanserin of QC sample in rat plasma (n = 6)

Analytes	Concentration added (ng/mL)	Intra-day precision			Inter-day precision			Recovery (%)	ME (%)
		Mean ± SD	RSD (%)	RE (%)	Mean ± SD	RSD (%)	RE (%)		
Flibanserin	800	747.3 ± 2.4	0.3	− 6.6	828.9 ± 81.8	9.9	3.6	91.5	0.92
	8000	8964.6 ± 1007.2	11.2	12.0	8426.0 ± 229.7	2.7	4.4	94.5	0.87
	80,000	83,660.1 ± 375.5	0.45	4.58	81,855.0 ± 1610.5	2.0	2.3	95.8	1.03

### Stability

The stability of the method was assessed the analytes under various temperature and time conditions (ambient temperature, 4 °C, after three freezing–thawing cycles and at − 20 °C). For this, the RSD and RE were utilized to evaluate the stability. The results of the stability tests are presented in Table 3. According to the RSD and RE results, the biases within the concentrations were all within the range of ± 15% of the nominal values. This indicated that flibanserin was stabilized in the plasma after being stored at an ambient temperature for 4 h, at 4 °C for 24 h, after three freeze–thaw cycles and at −20 °C for 30 days.

**Table 3:** Summary of stability of flibanserin in rat plasma under different storage conditions (n = 6)

Analytes	Concentration added (ng/mL)	Room temperature		4 °C		Three freeze–thaw		−20 °C	
		RE (%)	RSD (%)	RE (%)	RSD (%)	RE (%)	RSD (%)	RE (%)	RSD (%)
Flibanserin	800	− 13.2	8.6	3.2	4.4	7.9	7.3	10.8	7.9
	8000	− 4.7	7.0	7.9	6.0	8.3	6.5	− 9.9	5.3
	80,000	2.4	5.3	− 7.9	7.1	− 6.2	8.0	− 5.3	4.9

### Method application and pharmacokinetic study

**Fig. 3:**
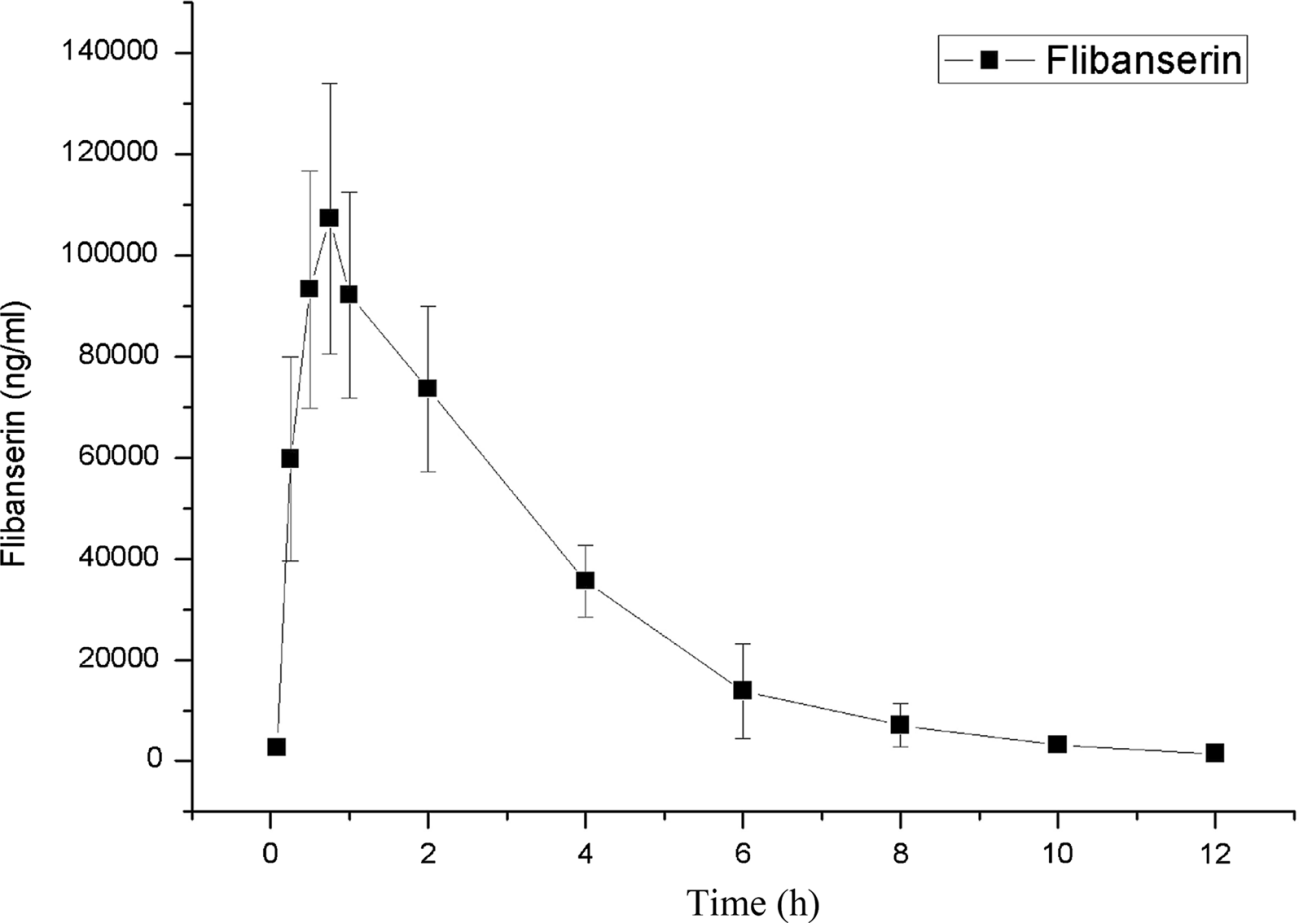
Mean plasma concentration–time curve after oral administration (10 mg/kg) of flibanserin

For the study of the pharmacokinetics, the current UPLC-MS/MS approach was applied efficiently for the determination of flibanserin in rat plasma at different time points. The blank rat plasma was utilized to dilute plasma samples when the analyte concentrations exceed the upper limit of the calibration curve. Figure 3 displays the curves of the mean flibanserin plasma concentration at different times after oral administration of flibanserin (10 mg/kg). We determined the pharmacokinetic parameters from the analysis of the non-compartmental mode. Table 4 shows the main plasma parameters that were measured. The pharmacokinetic data shows that after oral administration of flibanserin, the T_max_ and C_max_ were 0.79 ± 0.19 h and 108, 224.41 ± 25, 506.58 ng/mL, respectively. It can be observed that the plasma concentration of flibanserin increased rapidly initially. For the elimination of the analyte from the plasma, the t_1/2_ was determined to be 2.03 ± 0.66 h. The rapid decline in the plasma concentration indicates that the compound might be distributed into the target tissue quickly and for a short period of time. This leads to both a rapid therapeutic effect and a rapid onset of potential adverse reactions to the drug. So, users should notice the adverse effects of the drug in the initial period after its consumption [22, 23].

**Table 4:** The pharmacokinetic parameters of flibanserin in rat plasma after oral administration

Parameters	Unit	Mean po 10 mg/kg	SD	RSD/%
AUC_(0–t)_	μg/L h	351,658.00	77,499.85	22.0
AUC_(0–∞)_	μg/L h	356,517.60	77,670.82	21.8
MRT_(0–t)_	h	2.70	0.29	10.7
MRT_(0–∞)_	h	2.88	0.29	10.2
t_1/2_z	h	2.03	0.66	32.7
T_max_	h	0.79	0.19	23.7
V_z_/F	L/kg	0.09	0.04	42.5
CL_z_/F	L/h/kg	0.03	0.01	27.6
C_max_	μg/L	108,224.40	25,506.58	23.6

## Conclusion

In present study, an accurate, stable, sensitive and selective approach for the quantification of flibanserin in rat plasma was carried out and verified. This study is the first to report the determination of flibanserin in rat plasma by UHPLC-MS/MS. After optimization of the conditions, the method’s LLOQ was determined to be 100 ng/mL and the running time was 3 min. Finally, the UHPLC-MS/MS method was effectively applied for the study of the pharmacokinetics of flibanserin.

## Data Availability

All data and material analysed or generated during this investigation are included in this published article.
